# Vascularized Bone-Mimetic Hydrogel Constructs by 3D Bioprinting to Promote Osteogenesis and Angiogenesis

**DOI:** 10.3390/ijms20051096

**Published:** 2019-03-04

**Authors:** Takahisa Anada, Chi-Chun Pan, Alexander M. Stahl, Satomi Mori, Junji Fukuda, Osamu Suzuki, Yunzhi Yang

**Affiliations:** 1 Soft Materials Chemistry, Department of Applied Chemistry, Institute for Materials Chemistry and Engineering, Graduate School of Engineering, Kyushu University, 744 Motooka, Nishi-ku, Fukuoka 819-0395, Japan; 2 Division of Craniofacial Function Engineering, Tohoku University Graduate School of Dentistry, 4-1 Seiryo-machi, Aoba-ku, Sendai 980-8575, Japan; 3 Department of Orthopaedic Surgery, Stanford University School of Medicine, 300 Pasteur Drive, Stanford, CA 94305, USA; chichun@stanford.edu (C.-C.P.); amstahl@stanford.edu (A.M.S.); 4 Department of Mechanical Engineering, Stanford University School of Engineering, Building 380, Sloan Mathematical Center, Stanford, CA 94305, USA; 5 Department of Chemistry, Stanford University, Stanford, CA 94305, USA; 6 Graduate School of Dentistry, Tohoku University, 4-1 Seiryo-machi, Aoba-ku, Sendai 980-8575, Japan; satomi.mori.p7@dc.tohoku.ac.jp; 7 Faculty of Engineering, Yokohama National University, 79-5 Tokiwadai, Hodogaya-ku, Yokohama 240-8501, Japan; fukuda@ynu.ac.jp; 8 Department of Materials Science and Engineering, Stanford University, 300 Pasteur Drive, Stanford, CA 94305, USA; 9 Department of Bioengineering, 443 Via Ortega, Stanford University School of Engineering, Stanford, CA 94305, USA

**Keywords:** 3D bioprinting, gelatin methacrylate (GelMA), octacalcium phosphate (OCP), spheroids

## Abstract

Bone is a highly vascularized tissue with a unique and complex structure. Long bone consists of a peripheral cortical shell containing a network of channels for vascular penetration and an inner highly vascularized bone marrow space. Bioprinting is a powerful tool to enable rapid and precise spatial patterning of cells and biomaterials. Here we developed a two-step digital light processing technique to fabricate a bone-mimetic 3D hydrogel construct based on octacalcium phosphate (OCP), spheroids of human umbilical vein endothelial cells (HUVEC), and gelatin methacrylate (GelMA) hydrogels. The bone-mimetic 3D hydrogel construct was designed to consist of a peripheral OCP-containing GelMA ring to mimic the cortical shell, and a central GelMA ring containing HUVEC spheroids to mimic the bone marrow space. We further demonstrate that OCP, which is evenly embedded in the GelMA, stimulates the osteoblastic differentiation of mesenchymal stem cells. We refined the design of a spheroid culture device to facilitate the rapid formation of a large number of HUVEC spheroids, which were embedded into different concentrations of GelMA hydrogels. It is shown that the concentration of GelMA modulates the extent of formation of the capillary-like structures originating from the HUVEC spheroids. This cell-loaded hydrogel-based bone construct with a biomimetic dual ring structure can be potentially used for bone tissue engineering.

## 1. Introduction

Large bone defects caused by trauma, tumor resection and osteomyelitis have difficulty healing spontaneously. Biomaterials can be used for the treatment of bone defects instead of an autogenous bone graft. However, when bone substitute materials are implanted in large bone defects, insufficient vascularization often causes poor bone regeneration, because bone is a highly vascularized tissue [1]. Achieving early blood vessel formation in large bone grafts to supply nutrition and oxygen still presents a challenge. As the growth of blood vessels in bone and osteogenesis are coupled [2,3], many groups reported prevascularization strategies for bone substitutes using growth factors [4,5], and endothelial cells [6,7]. 

Bioprinting, which can build three-dimensional (3D) constructs using cells, proteins and biomaterials, is expected to be a powerful tool for the field of bone tissue engineering [8]. Stereolithography (SLA), and particularly digital light processing (DLP)-based SLA, is ideal for constructing 3D cell-laden hydrogels, because the method can reduce cell damage by the use of visible light and rapid production compared with laser-based SLA techniques [9]. We have already shown that the DLP-based SLA is a very useful technique for the fabrication of the 3D bifurcating tubular structures consisting of cell-laden hydrogels [10]. 

Various calcium phosphates have been utilized as bone substitute materials instead of autografts [11]. Among them, hydroxyapatite (HA) and biodegradable β-tricalcium phosphate (β-TCP) have been most widely used clinically. Octacalcium phosphate (OCP) is a biodegradable material like β-TCP [12,13]. OCP has been proposed to be a precursor to bone and tooth apatite crystals [14,15]. The transition of OCP to HA is thermodynamically favored under physiological conditions. We have been investigating the applicability of synthetic OCP as a bone substitute [16]. We have already revealed that OCP promotes osteoblastic [17,18], osteoclastic differentiation [19], and angiogenesis [20], so consequently, OCP promotes bone formation. We have proposed that OCP-apatite conversion is involved in these stimulatory effects of OCP [21,22].

Spherical cell aggregates known as spheroids mimic the functions of living tissues better than a monolayer culture. We previously fabricated spheroid culture chips [23] that can produce high numbers of the spheroids (about 500 spheroids per device) at a time and enable spheroids to be collected easily and non-invasively. We demonstrated that 3D culture using the spheroid culture device is useful for the maintenance of the function of hepatocytes [23] and the osteoblastic differentiation of mesenchymal stem cells [24]. It is reported that spheroid culture of endothelial cells promotes blood vessel formation in vitro and in vivo [25].

Regeneration of the large defects of bones or other organs needs rapid vascularization to supply oxygen and nutrients. In order to form new blood vessels in the scaffold, we previously developed several approaches to induce vascularization and bone formation by the combination of hydrogel and calcium phosphate or polycaprolactone struts [26,27]. In the present study, we developed the 3D bioprinting technique by DLP-based SLA for the precise positioning of 3D cultured cells and calcium phosphate materials into gelatin methacrylate (GelMA) hydrogels for bone tissue regeneration. The aim of this study was to develop the 3D hydrogel constructs containing calcium phosphate materials to improve the bone formation, and 3D cultured endothelial cells to facilitate the blood vessel formation. The construct was designed to mimic the bone formation process that naturally occurs in the body.

## 2. Results

### 2.1. Fabrication and Concept of 3D Hydrogel Constructs

**Figure 1:**
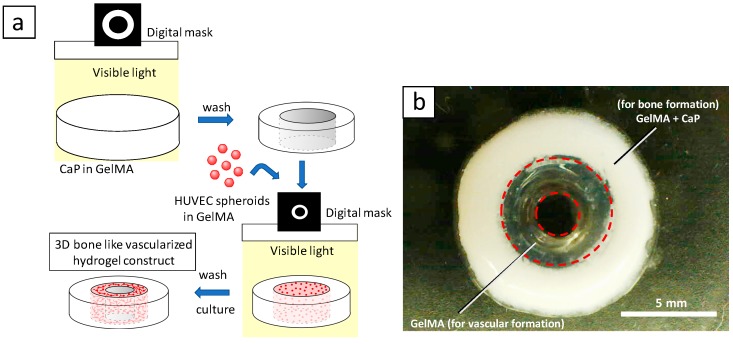
(**a**) Schematic illustration of fabrication process for 3D hydrogel constructs. (**b**) A photograph of 3D hydrogel constructs for vascular and bone formation. Bar = 5 mm.

Figure 1 shows the fabrication process and design concept of the 3D hydrogel constructs. Digital light processing enables precise positioning of hydrogels in the absence or presence of calcium phosphate materials (CaPs) with controlled spatial distribution (Figure 1a). GelMA was crosslinked by visible light using lithium phenyl-2,4,6-trimethylbenzoylphosphinate (LAP) as a photoinitiator. Figure 1b shows a photograph of 3D constructs. We designed the outer layer of the constructs to consist of GelMA containing CaPs and osteogenic differentiation medium for bone regeneration, and the inner layer of the constructs consists of GelMA containing angiogenic differentiation medium with HUVEC spheroids for vascular formation. 

### 2.2. Effect of OCP on the Osteoblastic Differentiation of Mesenchymal Stem Cells (MSCs) 

**Figure 2:**
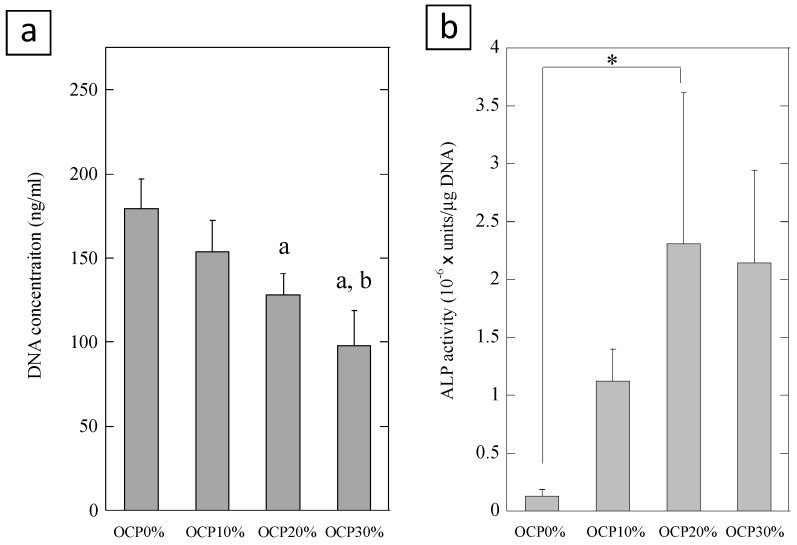
Cell proliferation (**a**) and alkaline phosphatase (ALP) activity (**b**) of C3H10T1/2 cells cultured on the GelMA hydrogel containing different amounts of octacalcium phosphate (OCP) (0, 10%, 20%, 30%) at day 14. Error bars show standard deviation. ^a^ statistical significance *p* < 0.05 compared with OCP0%, ^b^ statistical significance *p* < 0.05 compared with OCP10%, * statistical significance *p* < 0.05, Tukey-Kramer test, n = 3.

We evaluated cell proliferation and alkaline phosphatase (ALP) activity of mouse multipotent mesenchymal C3H10T1/2 cells cultured on the 7.5% GelMA containing different amounts of OCP (0, 10%, 20%, and 30%) (Figure 2). OCP resulted in significant decreases in the cell proliferation compared to in the absence of OCP. Cell proliferation was decreased with increasing amounts of OCP in the GelMA. When the cells were cultured on the GelMA containing OCP for 14 days, the level of ALP activity increased with increasing amounts of OCP up to 20% OCP. The activity of cells on the OCP20%-GelMA was significantly different compared to the OCP0%-GelMA (*p* < 0.05, Tukey-Kramer test, n = 3). These results suggest that GelMA containing OCP induces the differentiation of mesenchymal stem cells into osteoblastic cells.

### 2.3. Spheroid Culture of HUVEC 

**Figure 3:**
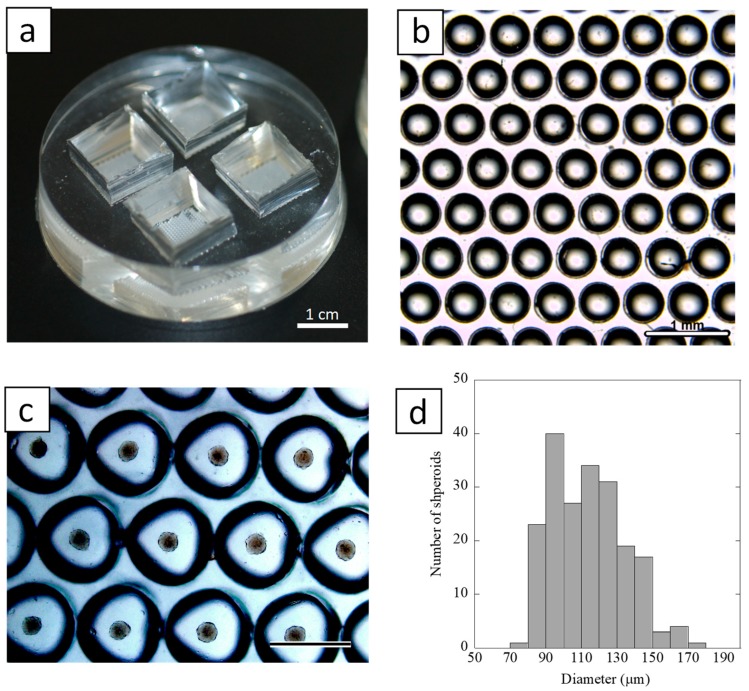
(**a**) A photograph of the spheroid culture chip. Bar = 1 cm. (**b**) Stereo microscopic image of the surface structure of the chip. Bar = 1 mm. (**c**) A light-microscopic image of spheroid formation on the spheroid culture chip at day 2. Bar = 500 μm. (**d**) Histogram of size distribution of spheroids formed on the culture chip at day 2 (n = 336).

Figure 3a shows a spheroid culture device which was made with poly(dimethylsiloxane) (PDMS). The chip was designed to be comprised of multicavities (512 wells, 0.5 mm in diameter (Figure 3b) in a triangular arrangement on a 12.5 mm × 12.5 mm section of the cell culture area. Figure 3c shows light-microscopic images of the spheroid formation of HUVEC on the culture device. It was observed that cells spontaneously dropped onto a hemispherical-shaped bottom of each cavity, and a single spheroid was formed in each cavity. The HUVEC inoculated into the chip formed spheroids within 3–4 h. The size distribution of the spheroids on day 2 in culture was shown in Figure 3d. The culture chip generated well-rounded spheroids with average diameters of 113.8 ± 20.7 μm (n = 336).

### 2.4. Effect of GelMA Concentration on the Sprout Formation from HUVEC Spheroids 

**Figure 4:**
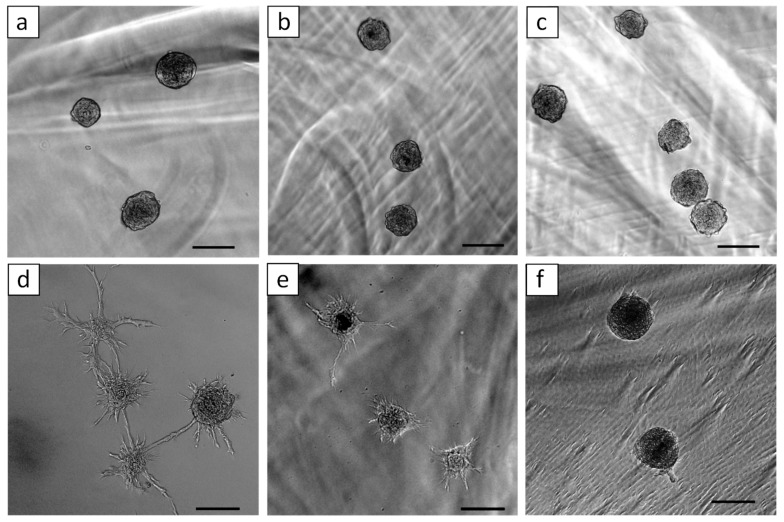
Three-dimensional in vitro angiogenesis of HUVEC spheroids in the different concentrations of GelMA ((**a**,**d**) 5%, (**b**,**e**) 7.5%, and (**c**,**f**) 10%) just after polymerization of GelMA (**a**–**c**), and after 1 day of culture (**d**–**f**). Bars = 100 μm.

**Figure 5:**
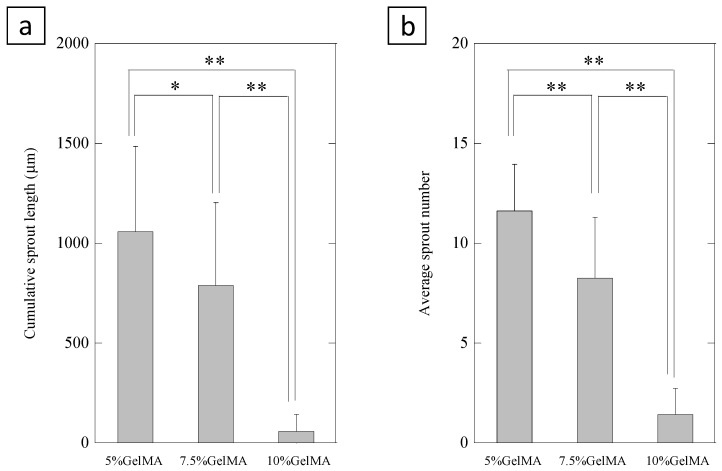
Cumulative sprout length (**a**) and average number (**b**) of capillary-like structures were measured by light microscopic images after 24 h. Error bars show standard deviation. * *p* < 0.05, ** *p* < 0.01, Tukey-Kramer test, n = 19–21 spheroids.

Figure 4 shows 3D in vitro angiogenesis of HUVEC spheroids which were embedded in different concentration of GelMA (5%, 7.5%, and 10%) hydrogel. HUVEC spheroids formed capillary-like structures in the GelMA hydrogel after 1 day of culture. We found that sprout formations from HUVEC spheroids were affected by the concentration of GelMA. We evaluated cumulative sprout length (Figure 5a) and the average number (Figure 5b) of capillary-like structures originating from the HUVEC spheroids in the different concentrations of GelMA. The quantitative analysis demonstrated that higher concentration of GelMA suppressed sprout formation. 

## 3. Discussion

We used GelMA as a matrix to retain OCP for stimulating the osteogenesis and to form blood vessel-like structures by endothelial cells. GelMA is a biodegradable, non-cytotoxic polymer, and has a property of promoting cell adhesion due to the cell binding motifs of gelatin [28]. In this work, GelMA was crosslinked by visible light using LAP as a photoinitiator. This crosslinking method is more suitable to encapsulate cells in the hydrogels compared to methods using the most common UV light photoinitiator, Irgacure 2959 (2-hydroxy-4′-(2-hydroxyethoxy)-2-methylpropiophenone), because UV light has been shown to have the potential to damage the encapsulated cells [29]. The advantage of SLA-based 3D fabrication in this study is its capacity to make two different cell culture environments within a single hydrogel—that is, the method offers appropriate environments for both osteogenesis and angiogenesis within the hydrogel through the spatial control of photocrosslinking of two different prepolymer solutions. We designed the outer layer of the constructs to contain osteogenic differentiation medium and OCP in the gel, while the inner layer of the constructs contained angiogenic differentiation medium in the gel.

We previously reported that OCP enhances the differentiation of mouse bone marrow-derived stromal cells to osteoblastic cells with upregulated osteogenic differentiation markers such as ALP, collagen 1a, and osterix [17]. OCP tends to hydrolyze to Ca-deficient HA, which is accompanied by an uptake of calcium ions from the surrounding solution, and the release of inorganic phosphate ions. It is probable that OCP hydrolysis is involved in acquiring the stimulatory capacity to activate bone tissue-related cells [17,18]. The results in the present study demonstrate that OCP embedded in GelMA hydrogels stimulated the osteoblastic differentiation of stem cells. Furthermore, both in vivo and in vitro experiments showed that OCP has a positive effect on angiogenesis during bone regeneration [20].

Numerous groups have reported blood vessel formation and regeneration using various hydrogels such as Matrigel [30], fibrin gel [31], and GelMA [32]. Spheroid culture of endothelial cells offers a useful approach to study angiogenesis, to analyze effects of drugs, [33,34], and to apply in tissue engineering [25]. We have developed spheroid culture devices which enable the production of a large number of spheroids with narrow size distribution [23]. In the present work, we tuned the diameter of the microwell (500 μm), and arranged 4 culture areas in a chip, which allows us to more easily optimize the culture conditions. One of the most important problems of spheroid culture is hypoxia in the core of the spheroids and subsequent central necrosis if the spheroids are applied to regenerative medicine. We reported that the spheroid culture chip can prevent the central hypoxia and necrosis of the spheroids [23,24], although we did not check the central necrosis of spheroids in this work due to the short culture time and relatively small size of the spheroids. We found that the GelMA concentration is critical for capillary sprouting originating from GelMA-embedded spheroids. Schurman et al. reported that the mechanical properties of GelMA hydrogels are related to the concentration of GelMA according to a power law [35]. In the present study, HUVEC favored the formation of 3D capillary networks in softer GelMA hydrogels, which is consistent with previous reports [32,36,37]. We expect that if the construct is implanted in bone defects, prevascularized structures originated from HUVEC spheroids would promote subsequent bone tissue repair, because reestablishment of the local circulation is an early event in bone fracture healing [38]. After vascular formation, migrated MSCs or pre-osteoblasts from host tissues would differentiate into osteoblasts by the effect of OCP. 

## 4. Materials and Methods

### 4.1. Synthesis of Gelatin Methacrylate (GelMA)

Gelatin methacrylate (GelMA) was synthesized as described previously [32]. Briefly, 10% type A gelatin (Sigma-Aldrich, St. Louis, MO, USA) was dissolved in phosphate buffered saline (PBS) at 60 °C. Methacrylic anhydride (8%) (Sigma-Aldrich) was added drop by drop to the gelatin solution and reacted at 50 °C for 3 h. Then, the solutions were dialyzed against distilled water by using 12–14 kDa cutoff dialysis tubing water at 40 °C for one week. The solution was lyophilized for 4 days.

### 4.2. 3D Printing

Octacalcium phosphate (OCP) was synthesized by direct precipitation according to a previously reported method [13]. OCP granules were obtained by passing through a standard testing sieve (270-mesh sieve and 53 μm). GelMA (7.5% w/v), OCP (5% w/v), a lithium phenyl-2,4,6-trimethylbenzoylphosphinate (LAP, Tokyo Chemical Industry Co., Ltd. Tokyo, Japan) (1% w/v) were mixed in DMEM medium (high glucose, L-glutamine). Hydrogels (d = 10.0 mm, h = 0.5 mm) were fabricated using an in-house custom-designed visible light projection SLA (3200 lumens) 3D printer [10,27]. The exposure time was 120 s. For SLA fabrication, the digital 3D model of the hydrogels and digital photomasks were designed using SolidWorks CAD software and was converted into printing instructions using free Slic3r software (Slic3r.org). After the first photopolymerization, the hydrogel was washed several times by PBS to remove the uncross-linked GelMA. Then, the EBM-2 medium containing GelMA (7.5% w/v) and the LAP photoinitiator (0.5% w/v) were added into the inner side of the hydrogel. The second photopolymerization with digital photomask was carried out by visible light (120 s), and washed several times by PBS.

### 4.3. Cell Culture

Multipotent mouse C3H10T1/2 cells (ATTC, Manassas, VA, USA) [39] were grown in DMEM media (Thermo Fisher, Carlsbad, CA, USA), supplemented with 10% fetal bovine serum (FBS; Life Technologies, Carlsbad, CA, USA) and 1% penicillin-streptomycin (PS; Thermo Fisher, Carlsbad, CA, USA). All cells were kept at 37 °C, 5% CO_2_ in a humidified incubator. The cells were passaged using trypsin-EDTA once per week and were used within 10 passages. Immortalized HUVECs constitutively expressing green fluorescent protein, a generous gift from Dr. J. Folkman (Boston Children’s Hospital, Boston, MA, USA) were maintained in an endothelial cell basal medium (EBM-2; Lonza) supplemented with endothelial growth BulletKit (EGM-2; Lonza) at 37 °C, 5% CO_2_ in a humidified incubator. The cells were passaged once per week.

### 4.4. Osteoblastic Differentiation of C3H10T1/2 Cells

A solution (100 μL) containing GelMA (7.5% w/v), different amounts of OCP (0, 10, 20, 30% w/v), and the LAP photoinitiator (0.1% w/v) in the osteogenic differentiation medium (DMEM supplemented with 10% FBS, 1% PS, 50 μg/mL ascorbate 2-phosphate, 10 mM β-glycerophosphate, and 100 nM dexamethasone) were added into 96-well plates (Corning). Photopolymerization was carried out by a digital light processing (DLP) projector (Vivitek, Fremont, CA, USA) for 5 min. C3H10T1/2 cells were seeded on the GelMA hydrogel with or without OCP at a cell density of 4 × 10^3^ cells/well in 96-well plates with 100 μL osteogenic differentiation medium. Cells were cultured for 14 days at 37 °C, 5% CO_2_ in humidified incubators. The culture medium was changed every two days. After 14 days of culture, cells were immersed in 0.1 mL of 0.2% Triton X-100 solution and sonicated on an ice bath. Then, cell lysates were centrifuged at 5000× *g* for 5 min to separate from the gel. DNA concentration of the supernatant was measured using a Quant-iT PicoGreen dsDNAkit (Thermo Fisher). Alkaline phosphatase (ALP) activity was measured using a LabAssay ALP kit (Wako Pure Chemical Industries, Ltd., Osaka, Japan). The ALP activity was normalized using DNA amounts as determined with the Pico Green kit. 

### 4.5. Fabrication of Spheroid Culture Chips and HUVEC Culture

We prepared a spheroid culture chip as previously reported [23]. The spheroid culture chip was designed to be comprised of multicavities (512 wells, 500 μm in diameter) in a triangular arrangement on a 12.5 mm × 12.5 mm section of the cell culture area. The chips were sterilized by autoclave at 121 °C for 20 min, then dried in an oven at 160 °C for 2 h. Before use, the chips were incubated with 1 mL of 4% Pluronic F-127 solution for 6 h. The polymer is adsorbed on the surface of the PDMS and prevents cell attachment [23]. The chips were then rinsed three times with EBM-2 to remove excess Pluronic F-127. HUVEC (25 × 10^4^ cells/mL) was added to the cell culture area of chips. After 2 days of culture, spheroids were photographed with a photomicroscope (Leica DFC300 FX, LeicaMicrosystems Japan, Tokyo, Japan). Twenty pictures from random areas of three different culture devices were taken. Spheroid diameters were analyzed using an image analysis program for Windows (Image-ProPlus 7.0, Media Cybernetics Inc., Bethesda, MD, USA). Diameters of 336 spheroids were measured. Spheroid diameter was defined as the average length of diameters measured at two-degree intervals joining two outline points and passing through the centroid. Spheroids were retrieved from culture chips by washing them out with PBS using a pipette. The collected spheroids were suspended in different concentrations of GelMA (5%, 7.5%, and 10%) solutions containing the LAP photoinitiator (0.2%). The suspension was added into a 48-well plate (400 μL/well). Photopolymerization was carried out by visible light projector for 20 min. EBM-2 (500 μL) was added into each well and cultured. After 24 h, cumulative sprout length and average number of capillary-like structures from the spheroids (n = 19–21) were measured and analyzed using an image analysis program (Image-ProPlus 7.0). 

### 4.6. Statistical Analysis

Results were expressed as the mean ± standard deviation (SD). All experiments were performed at least three times and showed reliable reproducibility. Data were analyzed by one-way analysis of variance (ANOVA) with the Tukey–Kramer multiple comparison analysis. A value of *p* < 0.05 was regarded as statistically significant.

## 5. Conclusions

In the present study, we have shown SLA-based fabrication of hydrogel constructs containing CaPs and HUVEC spheroids. OCP in the GelMA hydrogel stimulated the osteoblastic differentiation of MSCs. HUVEC spheroids, which were generated using our original spheroid culture device, formed capillary-like networks in GelMA hydrogels. Consequently, the hydrogel constructs could promote both osteoblastic and angiogenic differentiation. Even though in vivo evaluation and refinement of the construct components is necessary in the future, we believe that the endothelial cell-laden 3D hydrogel constructs containing calcium phosphate materials that stimulate osteoblastic differentiation have potential for use in bone tissue engineering.
